# Assessment of different continence definitions in the context of the randomized multicenter prospective LAP-01 trial—Does the best definition change over time?

**DOI:** 10.1186/s40001-024-01662-5

**Published:** 2024-01-18

**Authors:** Sigrun Holze, Anna Sophie Kuntze, Meinhard Mende, Petra Neuhaus, Michael C. Truss, Hoang Minh Do, Anja Dietel, Toni Franz, Jens-Uwe Stolzenburg

**Affiliations:** 1 Department of Urology, University of Leipzig, Liebigstraße 20, 04103 Leipzig, Germany; 2 Clinical Trial Centre Leipzig, University of Leipzig, Härtelstraße 16–18, 04107 Leipzig, Germany; 3 Institute for Medical Informatics, Statistics and Epidemiology, University of Leipzig, Härtelstraße 16–18, 04107 Leipzig, Germany; 4 Department of Urology, Klinikum Dortmund, Beurhausstr. 40, 44137 Dortmund, Germany

**Keywords:** Prostate cancer, Urinary incontinence, Prostatectomy, Continence definition, Pad usage

## Abstract

**Background:**

A uniform definition of continence is urgently needed to allow the comparison of study results and to estimate patient outcomes after radical prostatectomy (RP). To identify a practical definition that includes both objective and subjective aspects in a tangible way, we assessed different continence definitions and evaluated which best reflects the patients’ subjective perception of continence.

**Methods:**

Our analyses included 718 patients that underwent either robot-assisted radical prostatectomy (RARP) or laparoscopic radical prostatectomy (LRP) in a multicenter randomized patient-blinded trial. Continence was assessed through patient questionnaires prior to and at 3, 6 and 12 months after surgery which included the number of pads used per day, the ICIQ-SF and the question “Do you suffer from incontinence? (yes/no)” to assess subjective continence. We used Krippendorff’s Alpha to calculate the agreement of different continence definitions with the subjective perception.

**Results:**

At 3 months, the “0/safety pad” definition shows the highest agreement by alpha = 0.70 (vs. 0.63 for “0 pads” and 0.37 for “0–1 pad”). At 6 and 12 months “0 pads” is the better match, with alpha values of 0.69 (vs. 0.62 and 0.31) after 6 months and 0.70 (vs. 0.65 and 0.32) after 12 months. The ICIQ-SF score shows good correlation with the subjective continence at 3 months (alpha = − 0.79), the coefficient then decreasing to − 0.69 and − 0.59 at 6 and 12 months.

**Conclusion:**

The best continence definition according to the patients’ perspective changes over time, “0 pads” being the superior criterion in the long-term. We recommend using the 0-pad definition for standardized continence reporting, as it is simple yet as accurate as possible given the inevitably high subjectivity of continence perception.

*Trial registration* The LAP-01 trial was registered with the U.S. National Library of Medicine clinical trial registry (clinicaltrials.gov), NCT number: NCT03682146, and with the German Clinical Trial registry (Deutsches Register Klinischer Studien), DRKS ID number: DRKS00007138

**Supplementary Information:**

The online version contains supplementary material available at 10.1186/s40001-024-01662-5.

## Background

The carcinoma of the prostate is the most common cancer among European males, with an estimate of 470,000 cases in 2020 [1]. Radical prostatectomy (RP) as a typical treatment has evolved over the past decades, nowadays offering a high standard of therapy through minimally invasive and robot-assisted techniques [2–4]. Unfortunately, urinary incontinence (UI) is still a common side effect that negatively impacts the patients’ quality of life [2, 5–9].

Reporting continence rates is essential for measuring the success of refined surgery techniques, for comparing different study results and for providing a realistic estimate of the expected patient outcomes [10, 11]. The prevalence of post-prostatectomy incontinence (PPI) is influenced by clinical patient characteristics such as age, BMI and Gleason score of the tumor, as well as perioperative factors, for example surgeon experience, nerve-sparing approach, pelvic lymph node dissection and post-surgical measures such as rehabilitation and pelvic floor training [3, 12–16].

The biggest influence on reported incidences of PPI, however, is the absence of a single standardized definition of continence [6, 15]. In fact, a systematic review conducted by Borregales et al. showed that in the fourteen articles included in the study, nine different continence definitions were used [11]. The International Continence Society (ICS) has found continence rates ranging from 43 to 98% [14], which are additionally due to differences in data collection and assessment methods, length of follow-up and a divergence between patient and physician perception. Commonly discussed tools to objectively specify and quantify PPI are pad usage, pad weight tests and validated questionnaires, each characterized by a different set of advantages and disadvantages [7, 10, 11, 17, 18].

It is undisputable that a uniform continence definition which can serve as a standardized endpoint for studies on RP is urgently needed. The main challenge we face is that this “perfect” continence criterion should be methodically simple and, therefore, easily replicable, while including different aspects of UI to reach the highest possible level of accuracy. This paper aims to identify the definition that comes closest to the above criteria by evaluating which one matches best with the patients’ subjective perception of continence.

## Materials and methods

### Study design and data collection

The LAP-01 trial is a randomized, multicenter, patient-blinded controlled study to compare robot-assisted radical prostatectomy (RARP) and conventional laparoscopic radical prostatectomy (LRP). Between November 2014 and April 2019, 782 patients with a localized carcinoma of the prostate were recruited from four high-volume centers in Germany. The study subjects were randomized in a 3:1 ratio to undergo either RARP or LRP. The primary endpoint was defined as continence recovery at 3 months after removal of the urinary catheter. Secondary endpoints included potency, continence, clinical and oncological outcomes, quality of life and patient satisfaction up to 12 months post RP. A detailed description of the study design and procedures has been previously published [3]. The trial was approved by the ethical committees of all four participating centers. Written informed consent was obtained by all patients.

Continence data were gathered from patient questionnaires that were sent via mail to be filled out independently prior to and at 3, 6 and 12 months after surgery. To evaluate continence in three different categories (objective, subjective and symptom-based), we assessed: (1) the number of pads used per day; (2) the subjective continence using the question “Do you suffer from incontinence? (yes/no)” and (3) continence via the German version of the International Consultation on Incontinence Questionnaire Short-Form (ICIQ-SF). The ICIQ-SF consists of four questions: (1) How often do you leak urine?; (2) How much urine do you usually leak?; (3) How much does leaking urine overall interfere with your life? and (4) When does urine leak?, of which the first three are combined into a sum score ranging from 0 to 21 points, as proposed by Avery et al. [19]. Possible answers for the daily pad count were 0 pads, a safety pad, 1 pad, etc. up to 6 (or more) pads per day. If choosing “safety pad”, the patient also had to indicate whether the pad stayed dry or not. Clinical and socio-demographic data were retrieved from the patients’ medical records and case report forms.

### Statistical methods

Basis of our analyses is the Full Analysis Set (FAS) defined in the primary analysis of the trial [3], comprising all patients with valid continence information at 3 months post-surgery. We described the cohort by statistical standard parameters: Mean (standard deviation, SD) for continuous, absolute and relative frequencies for categorical variables. For skew distributed parameters, median and quartiles were applied.

To measure the agreement of different continence criteria we chose Krippendorff’s Alpha as appropriate measure of interrater agreement [20]. This parameter works in the presence of missing values. Alpha can be calculated for the agreement of the binary subjective continence with the binary criteria based on pads and for concordance with the continuous ICIQ sum. A SPSS macro written by A. F. Hayes calculated alpha and 95% confidence intervals by means of the bootstrap method (using *n* = 10,000 replications for binary, 2000 replications for ordinary variables) [21]. Krippendorff suggests alpha ≥ 0.667 as acceptable level of agreement [20]. We applied Alpha in two directions: We calculated the agreement between the criteria from documented pad use and the subjective continence. Then, we checked how well this criterion is reflected by concordant ICIQ sums.

The statistical analyses were performed by IBM SPSS Statistics, version 26. Additional analyses were done and graphs were generated by R including the packages *foreign*, *dplyr* and PropCIs [22–24].

## Results

### Baseline characteristics

Our study cohort consists of the FAS of 718 patients established by Stolzenburg et al. in the original analysis of the LAP-01 trial [3]. The sociodemographic and clinical characteristics are presented in Table 1. The study population showed a mean age of 64 years and a mean BMI of 27.4 kg/m^2^ upon admission to surgery. 96.8% of the patients used no pads prior to RP, fifteen patients used a safety pad and seven patients used one pad or more. Most of the patients (83.7%) had an intermediate- or high-grade tumor (Gleason ≥ 7). Five hundred-thirty patients were treated by RARP, while 188 patients were operated by LRP. Four hundred-forty patients (61.3%) received a nerve-sparing procedure, of which 372 procedures were bilateral.

**Table 1:** Baseline characteristics of the study cohort (*n* = 718)

Feature	Mean ± SD/Median [IQR]/*n* (%)
*Socio-demographic data*	
Age at surgery [y]	64.2 ± 6.7
Body size [cm]	177 ± 6
Body weight [kg]	85.8 ± 11.9
BMI [kg/m^2^]	27.4 ± 3.2
Karnofsky Index [%]	99 ± 4
Diabetes mellitus	101 (14.1%)
*Urinary tract medical history*	
History of urinary tract infection	22 (3.1%)
Transurethral resection of bladder cancer	2 (0.3%)
Transurethral resection of the prostate	19 (2.7%)
Other interventions on the urinary tract	168 (23.5%)
Incontinence: no. of used pads (pre op.)	
0	687 (96.8%)
Safety pad	15 (2.1%)
1 pad and more	7 (1.0%)
ICIQ sum (pre op.)	0.21 ± 0.84
*Surgical and oncological data*	
Diagnosis since [months]	2.1 [1.5, 3.0]
PSA pre op. [ng/ml]	7.81 [5.70, 11.9]
Prostate weight [g]	48 [38, 60]
Tumor stage	
pT1c	1 (0.1%)
pT2	453 (63.1%)
pT3	256 (35.7%)
pT4	5 (0.7%)
Gleason sum	
≤ 6	117 (16.3%)
7	473 (66.0%)
≥ 8	127 (17.7%)
Surgical approach	
RARP	530 (83.8%)
LRP	188 (26.2%)
Nerve sparing (realized)	
None	278 (38.7%)
Unilateral	68 (9.5%)
Bilateral	372 (51.8%)
Lymphadenectomy performed	546 (76.0%)
Duration of the op [min]	173 [145, 204]

### Continence rates at 3, 6 and 12 months

**Fig. 1:**
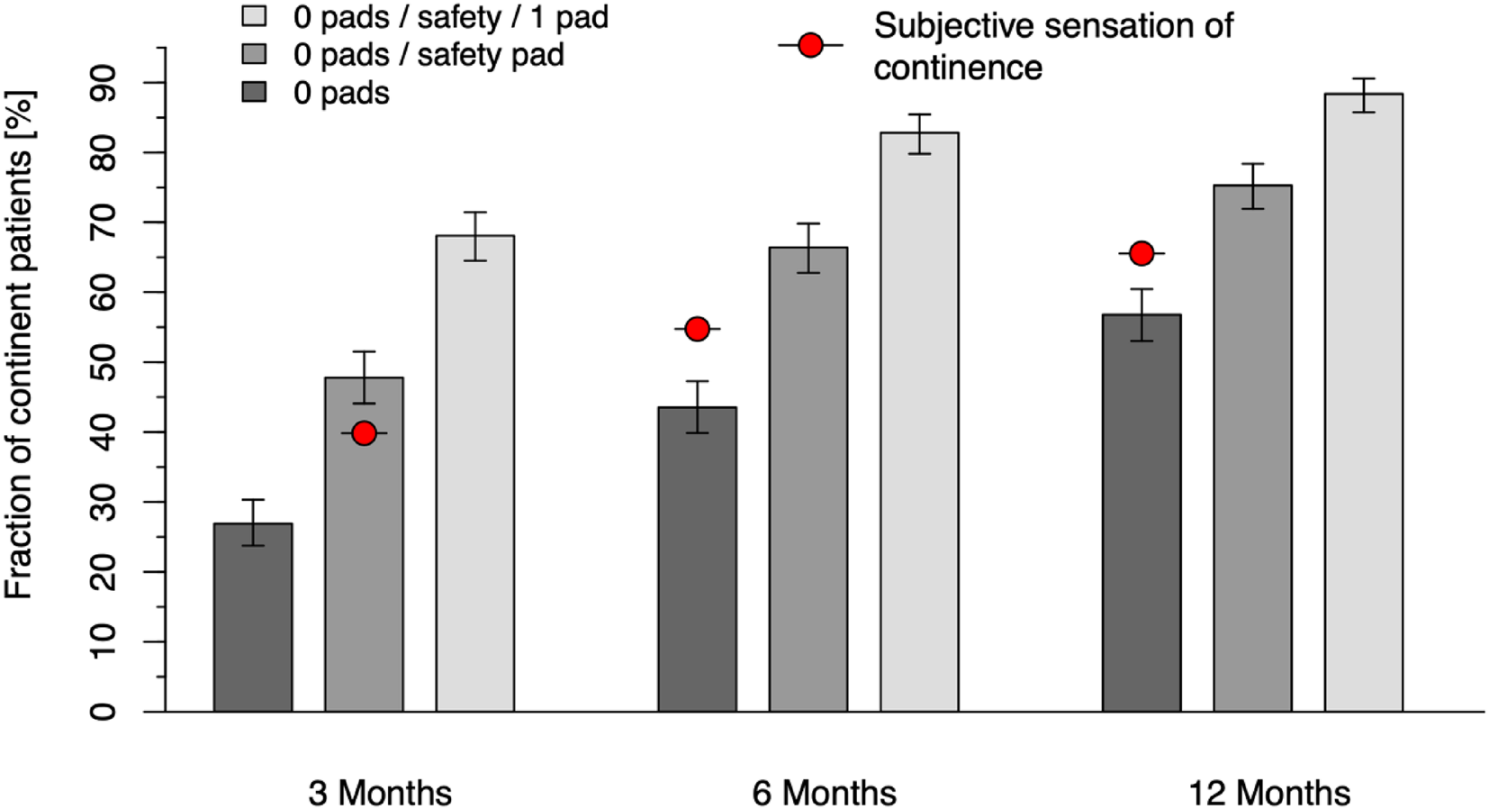
Continence rates at 3, 6 and 12 months by the definitions of 0 pads, 0/safety pad, 0–1 pad and subjective continence

Figure 1 displays the percentages of continent patients by different definitions based on the number of pads used in a 24-h period. The continence rate of patients using no pad or a single safety pad increased significantly from 47.8% at 3 months to 66.4% and 75.3% at 6 and 12 months. The percentage of patients using zero pads improved from 26.9% to 43.5 and 56.8% at 3, 6 and 12 months, respectively. Subjective continence according to the question “Do you suffer from incontinence? (yes/no)” is marked by the red dots, each placed over the criterion with which it best agrees at the different evaluation points (cf. Table 2). As per this self-assessment, continence rates are 39.8% at 3 months, 54.7% at 6 months and 65.5% at 12 months. The proportion of patients using up to 1 pad per day, often labeled as socially continent, is significantly higher than the before mentioned rates (68.1%, 82.8% and 88.4%).

**Table 2:** Agreement between the different criteria and subjective continence at 3, 6 and 12 months measured by Krippendorff’s Alpha

Criterion	Subjective perception of continence^a^		
	Krippendorff's Alpha [95% CI]		
	3 months	6 months	12 months
0 pads	0.63 [0.46, 0.80]	0.69 [0.54, 0.82]	0.70 [0.54, 0.83]
0-safety pad	0.70 [0.55, 0.84]	0.62 [0.46, 0,80]	0.65 [0.47, 0.81]
0–1 pad	0.37 [0.20, 0.56]	0.31 [0.09, 0.51]	0.32 [0.07, 0.55]
ICIQ-SF sum score^b^	− 0.79 [− 0.89, − 0.69]^c^	− 0.69 [− 0.79, − 0.58]^c^	− 0.59 [− 0.70, − 0.49]^c^

^a^According to the question "Do you suffer from incontinence? (yes/no)"

^b^ICIQ-SF sum score = *Q*1 + *Q*2 + *Q*3 (Avery et al.)

^c^Krippendorff's alpha can be used on ordinal variables. In this case, the more negative the values, the more continence is associated with a low score on the analog scale. If all continent subjects reported a 0 and all incontinent subjects reported higher values, we would have the ideal of − 1

### Association of subjective perception of continence and different definitions

To determine which of the pad criteria best reflects the patients’ perception of continence, we calculated Krippendorff’s Alpha as coefficient of agreement (Table 2). At 3 months, the highest agreement between subjective continence and number of pads is reached by the 0/safety pad definition with 0.70. At the 6- and 12-month evaluations, however, the 0-pad criterion replaces 0/safety pad as the best definition with alpha values of 0.69 and 0.70.

To illustrate this in absolute numbers, we cross-tabulated the patients’ subjective continence (in rows) and the different pad criteria (in columns) in Table 3. The counts of patients for whom the objective (pads) and subjective estimates agree are shown in the main diagonal, whereas counts in the secondary diagonal feature the discrepant patient answers. For example, at 3 months post-RP, 174 (93.5%) of the 186 patients classified as continent by the 0-pad definition also felt subjectively continent. Equally, 405 (79.9%) of the 507 incontinent patients according to this definition also perceived themselves as incontinent. Looking at the secondary diagonals, we find that with the 0-pad classification, 102 + 12 = 114 of 693 (16.5%) were determined as either continent or incontinent but did not feel that way. There are 24 + 79 = 103 of 693 (14.9%) discrepant estimates by the 0-safety pad definition and 10 + 206 = 216 of 693 (31.2%) by the 0–1 pad definition. The high number of discrepancies within the 0–1 pad classification matches the alpha values of 0.37 and lower found in the previous analysis (Table 2), therefore, declassifying the 0–1 pad definition at all points in time.

**Table 3:** Subjective continence and different definitions based on pad count in absolute numbers

3 months							
		0-pad definition		0-safety pad definition		0–1 pad definition	
Subjective continence ^*a*^		Continent	Incontinent	Continent	Incontinent	Continent	Incontinent
Continent	276 (39.8%)	174 (93.5%)	102 (20.1%)	252 (76.1%)	24 (6.6%)	266 (56.4%)	10 (4.5%)
Incontinent	417 (60.2%)	12 (6.5%)	405 (79.9%)	79 (23.9%)	338 (93.4%)	206 (43.6%)	211 (95.5%)
Total (100%)	693	186	507	331	362	472	221

6 months							
		0-pad definition		0-safety pad definition		0–1 pad definition	
Subjective continence ^*a*^		Continent	Incontinent	Continent	Incontinent	Continent	Incontinent
Continent	376 (54.7%)	284 (95.0%)	92 (23.7%)	354 (77.6%)	22 (9.5%)	370 (65.0%)	6 (5.1%)
Incontinent	311 (45.3%)	15 (5.0%)	296 (76.3%)	102 (22.4%)	209 (90.5%)	199 (35.0%)	112 (94.9%)
Total (100%)	687	299	388	456	231	569	118

12 months							
		0-pad definition		0-safety pad definition		0–1 pad definition	
subjective continence ^*a*^		Continent	Incontinent	Continent	Incontinent	Continent	Incontinent
Continent	445 (65.5%)	367 (95.1%)	78 (26.6%)	428 (83.8%)	17 (10.1%)	440 (73.3%)	5 (6.3%)
Incontinent	234 (34.5%)	19 (4.9%)	215 (73.4%)	83 (16.2%)	151 (89.9%)	160 (26.7%)	74 (93.7%)
Total (100%)	679	386	293	511	168	600	79

^a^According to the question “Do you suffer from incontinence?” (yes/no)

Furthermore, at 3 months, the alpha coefficient for the ICIQ-SF sum score is − 0.79 (Table 2), indicating better agreement with subjective continence than any definition by the number of pads. Despite this good concordance, the alpha values decrease to − 0.69 and − 0.59 over time, suggesting that the ICIQ-SF score loses significance in the long-term.

We applied Krippendorff's Alpha as a versatile measure of agreement. Weighted Kappa (Cohen) differed only few from Alpha but was not applicable for the ICIQ scale. An additional cross-tabulation of subjective continence and patient answers to the ICIQ-SF questions on amount and frequency of urine loss exemplified by the 3-month evaluation is displayed in Additional file 1: Table S1.

## Discussion

To date, the most frequently utilized method for continence assessment in RP patients is the number of pads used in a 24-h period, as it is easily accessible, objective and reliable in an ambulatory context [6, 9, 16, 25, 26]. However, there is little agreement on how this criterion should be applied. Some authors suggest to consider continent all patients who use up to one pad per day [6], whilst others find this “social continence” to be too lenient [5, 8, 16]. Our results are in accordance with the latter, showing that every 4th patient classified as continent by the one pad definition reports to suffer from incontinence. It is also possible to document the use of a safety pad in a separate category, which we consider highly relevant. Thereby, patients with no leakage using one pad for security reasons only can be distinguished from those having actual urine loss. Since the ICS defines incontinence as the “complaint of involuntary loss of urine” [27], one could argue to consider the safety pad group continent if the pad stays dry [6]. Contrastingly, Liss et al. found a remarkable decrease in patients’ quality of life when using even just a safety pad, hence they strongly suggest the strict definition of zero pads [5].

A common argument against the use of daily pad count is the higher accuracy and objectivity provided by pad weight protocols. They are the preferred tool when proposing surgical treatment of UI because operative outcomes largely depend on a particularly precise assessment of preoperative incontinence severity [7, 27, 28]. Unfortunately, pad weight tests involve logistical difficulties and require a high level of patient compliance which makes them inconvenient for daily use and large patient cohorts [9, 28, 29]. Patients with severe symptoms looking for UI treatment are likely to comply, however, consistent pad testing is not realistic if the continence assessment is used as a primary endpoint for a large-scale study on RP.

The goal of this study was to determine which definition based on pads per day best reflects the patients’ subjective perception of continence. Interestingly, we found that the definition that corresponds best to the subjective continence depends on the time after surgery. According to our analysis, the best criterion is 0/safety pad at 3 months, then switches to 0 pad at 12 months. This could possibly be a result of disparate patient expectations shortly after vs. 1 year after RP. Although urinary incontinence is known to be a common side effect of RP, it is often labeled a temporary problem [16]. Consequently, a patient using a security pad might consider himself continent at 3 months post-RP but may not tolerate a safety pad at 12 months after surgery. Another influencing factor could be the different level of physical activity at 3 months compared to 12 months post-surgery. Since PPI rates are subject to change within the first months before reaching a plateau after approximately a year [12, 25], we consider our 12-month evaluation the most relevant in terms of establishing a standardized continence definition. We identified 0 pads as the superior definition at this point in time.

While the 0-pad definition is valued for its objectivity and conclusiveness, a major point of criticism is a lack of accuracy regarding the different aspects of UI symptoms [8, 11, 17, 29]. Some authors argue that the use of zero pads does not necessarily correspond to complete urinary continence, because often times patients using no pads still report leakage [7, 8, 17, 29]. Validated questionnaires such as the ICIQ-SF reflect a more detailed image of the patients’ UI status, as they are able to capture frequency, amount and impact of urine loss on everyday life [19]. This, however, comes with the risk of over-reporting severity. Borges et al. recently reported that ICIQ-SF evaluation rated UI as severe for 80.6% of the patients, whilst only 20.6% perceived their UI as severe [18].

Looking at the association of subjective continence with ICIQ-SF scores in our cohort, we find a significant agreement at 3 months. At 12 months, however, the patients’ perceived continence was better reflected by the 0-pad definition than by the ICIQ-SF evaluation. We therefore assume that a detailed assessment via ICIQ-SF is reasonable in the beginning, but a simple and strict criterion is needed in the long term. The ICIQ-SF is excellent at detecting mild UI, which Azal et al. and Mata et al. believe to be the main source of discrepancies between ICIQ-SF results and number of pads used [25, 30]. Patients with slight leakage tend to not using any pads, possibly because they do not consider their UI as severe [25, 30]. Interestingly, in our study, 44% of the patients who felt subjectively continent at 3 months also reported leakage (cf. Additional file 1: Table S1). Furthermore, Cortés et al. found no difference in quality of life between patients with an ICIQ score of 0 compared to patients scoring 1 or 2 points for leaking “a small amount” or leaking “about once a week” [7, 19]. These findings indicate that patients possibly consider themselves continent, even if losing small amounts of urine. Based on this and the fact that we believe a uniform continence definition should be kept simple, we suggest using the 0-pad criterion, even though it may include some patients with minimal leakage.

Within the highly complex and subjective topic of continence evaluation there are certain limitations that should be acknowledged. Firstly, patient answers are largely influenced by each patients’ personality and individual characteristics [7, 28], which inevitably entails a loss of accuracy in continence reporting. Current research on post-RP continence assessment constantly aims to reduce inconsistencies to a minimum by capturing all different aspects in a very detailed manner. This proves to be very beneficial for the severely incontinent individual [28]. However, this effort which also comes with a risk of losing patient compliance might not be fitting for large patient groups that mainly show slight or no incontinence at all. A continence assessment that points the way for further treatment has different priorities than one aiming to set a primary endpoint for a large-scale study on RP. While the former rightfully prioritizes accuracy over convenience, the latter should be practical and simple while reflecting the patients’ real continence status as precisely as possible. Therefore, we find a small range of inconsistencies acceptable for the purpose of being able to compare study results in a simple and easily replicable way.

Secondly, we are aware that the single question “Do you suffer from incontinence” poses a very simplified approach to assessing subjective continence. However, we purposely decided on this method not to question or test existing validated instruments, but rather to identify where the patient stands amidst the many proposed measures and possible definitions. By including this simplified subjective assessment, we gain valuable information on how to best convert the many different aspects of UI into a simple classification that is still able to reflect the patients’ subjective perspective. We consider this a strength of our study that adds to previous findings on this topic.

Further strengths of our study include the nature and the time frame of the LAP-01 trial. It is the first multicenter, randomized, patient-blinded controlled study worldwide on functional and oncologic outcomes of RARP vs. LRP [3], providing an excellent context for evaluating different continence definitions. We assessed continence at 3, 6 and 12 months, enabling us to observe a development over time based on a large, randomized cohort. In combination with the added safety pad category, this gives us a much more detailed and in-depth approach to continence assessment through the daily pad count.

Future studies on this topic could be conducted to investigate whether subjective perception of UI differs between patients from various backgrounds.

## Conclusions

We aimed to identify a continence definition for the purpose of comparing study results on RP that is practical yet as accurate as possible given the inevitably high subjectivity of the topic. Our findings indicate that which definition best reflects the patients’ subjective continence depends on the time since surgery, “0 pads” corresponding best with patient perception in the long-term. Therefore, we suggest 0 pads as a standardized continence definition. We consider the ICIQ-SF a valuable additional tool for short-term continence evaluation.

### Supplementary Information

**Additional file 1: Table S1.** Patient answers to ICIQ-SF Q1 and Q2 by subjective continence at 3 months.

## Data Availability

The datasets used during the current study are available from the corresponding author on reasonable request.

## Abbreviations

RP: Radical prostatectomy
UI: Urinary incontinence
PPI: Post-prostatectomy incontinence
ICS: International Continence Society
RARP: Robot-assisted radical prostatectomy
LRP: Laparoscopic radical prostatectomy
ICIQ-SF: International Consultation on Incontinence Questionnaire Short-Form
FAS: Full analysis set
SD: Standard deviation
BMI: Body mass index
