# Supplementary material for: Assessment of different continence definitions in the context of the randomized multicenter prospective LAP-01 trial—Does the best definition change over time?
Source: Eur J Med Res. 2024 Jan 18;29:58. doi: 10.1186/s40001-024-01662-5 (PMC10795395; doi:10.1186/s40001-024-01662-5)
Supplement: Supplementary file 1 — Additional file 1: Table S1. Patient answers to ICIQ-SF Q1 and Q2 by subjective continence at 3 months. [file 40001_2024_1662_MOESM1_ESM.docx]

**Supplementary Table 1** Patient answers to ICIQ-SF Q1 and Q2 by subjective continence at 3 months

| **ICIQ-SF Q1: How often do you leak urine?** | | |  |  |  |
| --- | --- | --- | --- | --- | --- |
| **urine loss - frequency** | **subjective continence*** | |  |  |  |
|  | continent | incontinent |  |  |  |
| never | 154 (55.8%) | 3 (0.7%) |  |  |  |
| once weekly | 81 (29.3%) | 29 (7.0%) |  |  |  |
| twice-thrice weekly | 25 (9.1%) | 52 (12.5%) |  |  |  |
| once daily | 11 (4.0%) | 51 (12.3%) |  |  |  |
| repeatedly | 5 (1.8%) | 258 (62.3%) |  |  |  |
| permanently | 0 | 21 (5.1%) |  |  |  |
| total (100%) | 276 | 414 |  |  |  |
|  |  |  |  |  |  |
| **ICIQ-SF Q2: How much urine do you usually lose?** | | |  |  |  |
| **urine loss - quantity** | **subjective continence *^a^*** | |  |  |  |
|  | continent | incontinent |  |  |  |
| nothing | 159 (57.6%) | 4 (1.0%) |  |  |  |
| small amount | 116 (42.0%) | 334 (80.6%) |  |  |  |
| moderate amount | 1 (0.4%) | 58 (14.1%) |  |  |  |
| large amount | 0 | 15 (3.6%) |  |  |  |
| total (100%) | 276 | 411 |  |  |  |

*^a^ According to the question “Do you suffer from*
